# One-year evaluation of automated insulin delivery systems in adults with type 1 diabetes

**DOI:** 10.3389/fdgth.2025.1596188

**Published:** 2025-07-01

**Authors:** Pedro Pujante, Ana Victoria García, Elsa Villa-Fernández, Miguel García-Villarino, Tomás González-Vidal, Lucía Díaz-Naya, Gala Gutiérrez Buey, Brenda Veiguela, Diego Rivas-Otero, Edelmiro Menéndez-Torre, Elías Delgado, Jessica Ares, Carmen Lambert

**Affiliations:** ^1^ Department of Endocrinology and Nutrition, Hospital Universitario Central de Asturias, Oviedo, Spain; ^2^ Grupo de Endocrinología, Nutrición, Diabetes y Obesidad, Instituto de Investigación Sanitaria del Principado de Asturias (ISPA), Oviedo, Spain; ^3^ Department of Medicine, Universidad de Oviedo, Oviedo, Spain; ^4^ Department of Endocrinology and Nutrition, Hospital Universitario de Cabueñes, Gijón, Spain; ^5^ Centre for Biomedical Network Research on Rare Diseases (CIBERER), Instituto de Salud Carlos III, Madrid, Spain

**Keywords:** automated insulin delivery, type 1 diabetes, hybrid close loop, diabetes technology, insulin

## Abstract

**Introduction:**

This study evaluates the effectiveness of four commercially available automated insulin delivery (AID) systems in routine clinical practice for type 1 diabetes management and compares their first-year outcomes.

**Materials:**

This retrospective study (October 2020–October 2023) included glucometric data from type 1 diabetes patients collected from the Asturias Automatic Insulin Devices Registry. People with type 1 diabetes using four different AID systems [Minimed™780G (MM780G), Accu-Chek® Insight-Diabeloop (DBLG), Tandem-Control-IQ, and Ypsopump Cambridge HCL-app (Cam-APS)] were included in the study. Primary outcomes were glycaemic control after one year. Secondary outcomes compared the results between the four systems.

**Results:**

174 patients (60: MM780G, 45: DBLG, 30: Control-IQ, 39: Cam-APS), aged 18–73, predominantly women, were enrolled. At baseline, glycemic control, measured by the achievement of the international consensus targets [TBR (Time Below Range) <4%; TIR(Time in Range) >70% and TAR (Time Above Range) <25%] was only met by 9.72% of participants, while after 1 year of an AID system use, it improved to more than 52%. When comparing between systems, TIR improved significantly after 1 year in all systems. However, Control-IQ did not show improvement in mean glucose and Glucose management index (GMI) and only users of DBLG showed improvement in coefficient of variation (CV). MM780G users achieved the best results after 12 months in mean glucose, TIR and GMI. However, their baseline situation was also better than that of the other groups.

**Discussion:**

This study confirms that, after 12 months of routine clinical use, the use of commercially available automated insulin delivery (AID) systems significantly improves glycemic control in individuals with type 1 diabetes. However, the degree of improvement varies depending on the specific system used.

These findings underscore the importance of selecting and optimizing AID systems individually to maximize clinical benefits in type 1 diabetes management.

## Introduction

The use of technology applied to medicine is undergoing exponential growth and the management of type 1 diabetes (T1D) is no exception (1, 2). Automated insulin delivery (AID) systems, also known as advanced hybrid closed-loop systems, work by delivering subcutaneous insulin in an automatic way, based on the data from a Continous Glucose Monitor (CGM) system and its control algorithm. Therefore, AID systems automatically increase or decrease the basal insulin delivery based on sensor glucose levels. However, users still need to manually dose prandial insulin (1).

Various models of AID systems have been marketed, all of which have demonstrated the capability to enhance the management of T1D (3–6). Each AID is composed of 3 main elements: a CGM, an insulin pump and its own particular algorithm. Among the different AID systems used worldwide, the most common ones used in our country are the following (2): from Medtronic the MiniMed^TM^ 780G with SmartGuard^TM^ (MM780G; Medtronic, USA) (3), from Ypsomed the mylife YpsoPump with CamAPS FX algorithm (CamAPS; Ypsomed, UK) (4), from Tandem Diabetes the t:slim X2™ with Control-IQ algorithm (Tandem Diabetes; USA) (6) and from Roche the Accu-Check Insight with Diabeloop Generation 1 algorithm (DBLG; Roche, Switzerland) (5).

These systems have already shown a reduction in glycated hemoglobin (HbA1c) levels and an increase in time in range, surpassing other treatment alternatives (7) in children (8), adolescents (9), adults (10, 11) and pregnancy (12). Therefore, clinical practice guidelines recommend this treatment for most patients with T1D (13–15). Also, some studies have compared the effectiveness of different AID systems in adults with T1D (16–20). Two single-center studies compared the effectiveness of MM780G and Control-IQ (16, 17) and another study compared the effectiveness of MM780G, Control-IQ and DBLG (19). Anyway, results among these studies were inconsistent (16, 17, 19). The follow-up was longer than 6 months in only one of these studies (17). Additionally, the effectiveness of these AID systems may differ among them, and there is a need for studies comparing the long-term effectiveness of these systems. Besides, there is not a clear indication of which system is the most indicated for each patient. To the best of our knowledge, no studies have simultaneously compared the long-term glycaemic control achieved by using MM780G, CamAPS, DBLG and CamAPS.

Hence, the primary aim of this real-life study was to report the evolution of the different variables describing glucose control in the patients using AID system during one year follow-up (at baseline, 3 months, 6 months and 12 months) and the secondary aim was to compare the 1 year performance of the four different AID systems. The variables used for glucose control were mean TIR, TAR, TBR, GMI, and CV at different time points and the proportion of patients meeting the international targets for TIR>70%, TAR>25%, TBR<%, CV<36% at different time points.

## Methods

A retrospective study was performed from October 2020 to October 2023, including T1D patients who initiated insulin treatment with one of the four AID systems: Tandem t:slim X2 Control IQ™system (Tandem Control-IQ; Tandem Inc., San Diego, California), Accu-Chek® Insight with Diabeloop™ (DBLG; Roche, Basel, Switzerland) and Ypsopump Cambridge HCL app (Cam-APS, CamDiab, UK), all of them associated with Dexcom G6 (Dexcom Inc., San Diego, CA) system; Minimed™780G system (MM780G; Minimed Medtronic, Northridge, California), integrated with the Guardian Sensor 4 (Medtronic, Northridge, California);

All the patients were enrolled in the Asturias Automatic Insulin Devices Registry from Cabueñes University Hospital and the Central Asturias University Hospital. Only patients who had received treatment for more than one year were eligible for this study.

Written informed consent was obtained from all participants, at the moment of the AID system implementation and the study was conducted in accordance with the principles of the Declaration of Helsinki for human research. The protocol was approved by the Ethical Committee of the Central University Hospital of Asturias (Project No. 2023.463, Oviedo, Asturias, Spain).

Patients were selected according to the following criteria: (A) T1D diagnosis at least one year prior to the study, (B) insulin therapy with continuous subcutaneous insulin infusion (CSII) device or multiple dose injection (MDI) and (C) previous use of CGM (D) Patients with automatic mode less than 90% or with percentage of monitoring data information less than 70% were excluded. Patients with other types of diabetes or who had previously used an AID system were excluded, except for those with a predictive low glucose suspend system (PLGS; Medtronic 640G and Tandem t:slim X2 Basal IQ™ system). The choice of AID system was not randomized but was at the discretion of the endocrinologist based on individual patient characteristics. In no case did participation in this study benefit the waiting list for implementation of the AID system.

At baseline, the following data were collected from each patient: demographics (sex, age), diabetes duration, previous insulin therapy and HbA1c. Glucometrics data from the previous 14 days before the systems implementation, as well as at three, six and twelve months of follow up, were downloaded from the available web-based software respectively (Libreview for Freestyle Libre, Clarity for Dexcom, Carelink for Guardian, Yourloops for Diabeloop AID, and Glooko for Tandem and Ypsomed AID). Mean glucose, Glucose Management Indicator (GMI), coefficient of variation (CV), time in range (TIR, time with glucose values between 3.9 and 10.0 mmol/L), time above range 1 (TAR1, time with glucose values between 10.0 and 13.9 mmol/L), time above range 2 (TAR2, time with glucose values >13.9 mmol/L), and time below range 1 and 2 (TBR1, time with glucose values between 3.0 and 3.9 mmol/L; TBR2, time with glucose values <3.0 mmol/L) were analyzed, according to the International Consensus on Time in Range (21).

The primary outcomes were to analyze the differences in TIR in the whole cohort, from baseline to 3, 6 and 12 months of follow-up, and the difference in TIR between the four AID systems. Secondary outcomes were to evaluate the percentage of patients with TIR >70%, the proportion of patients with CV <36%, time in hypoglycemia and hyperglycemia derived from continuous glucose monitoring metrics as well as the overall targets from the International Consensus: TBR (time with glucose values <3.9 mmol/L) <4%; TIR >70% and TAR (time with glucose values >10.0 mmol/L) <25% (21), at 3, 6 and 12 months of follow-up in the all the patients as well as the differences between groups.

Statistical analysis was performed using JASP version 0.18.1.0 statistical software. A descriptive analysis of continuous variables was performed by calculating their mean and standard deviation. Categorical variables were expressed as percentages.

Data were analyzed using the Anova test to compare between systems, and the repeated measures ANCOVA test for comparison between time points, adding the basal glucose, the time of diabetes evolution and the previous use of an insulin pump as covariants. For categorical variables, the Cochran Q test and Friedman test were used. A *p*-value less than 0.05 was considered statistically significant.

## Results

### Impact of 1 year of implementation of an AID system

Demographic characteristics of participants are shown in Table 1. In brief, 174 participants with T1D, included in the Asturian Automatic Insulin Devices Registry, ranged in age from 18 to 73 years (median age of 45 years) and with a median disease duration of over 24 years. At baseline, the mean glucose level of patients was 8.81 mmol/L, the GMI was 53.9 ± 6.2 mmol/mol, the CV was 37.29%, the TIR was 64.22%, the TAR was 31.91%, and the TBR was 3.91% (Table 2). All participants completed at least 1 year with an AID system with the follow-up visits at 3, 6 and 12 months.

**Table 1:** Basal characteristics of patients.

Variable	All	MM780G	DLBG	Control-IQ	CAM-APS	*P* ^a^
	(*n* = 174)	(*n* = 60)	(*n* = 45)	(*n* = 30)	(*n* = 39)	
Age (years)	45 [18–73]	47 [18–68]	43 [31–71]	43 [25–73]	43 [18–69]	ns
Sex male (*n*, %)	56 (32.2%)	20 (33.3%)	13 (28.9%)	11 (36.7%)	14 (35.9%)	ns
Diabetes duration (years)	24.1 [2.7–55.5]	24.9 [2.9–55.5]	27.7 [2.7–53.6]	19.8 [1.3–57.1]	21.2 [8.2–47.5]	*
HbA1c (mmol/mol)	55 [38–85]	53 [38–72	56 [41–78]	52 [39–78]	58 [41–85]	ns
HbA1c <53mmol/mol (*n*, %)	74 (42.5%)	29 (48.3%)	17 (42.5%)	15 (50%)	13 (33.3%)	ns
GMI (mmol/mol)	53.9 ± 6.2	51.6 ± 3.4	55.2 ± 6.6	53.6 ± 6.1	56.8 ± 7.5	***
CV (%)	37.29 ± 6.00	34.49 ± 5.22	38.41 ± 5.91	36.53 ± 5.30	38.92 ± 6.79	*
TIR	64.22 ± 13.33	70.74 ± 8.59	58.84 ± 15.53	64.58 ± 13.71	60.56 ± 12.82	***
Previous treatment						
-MDI (*n*, %)	44 (25.3%)	8 (13.3%)	13 (28.9%)	7 (23.3%)	16 (41.0%)	***
-Insulin pump (*n*, %)	98 (56.3%)	33 (55%)	32 (71.1%)	11 (36.7%)	22 (56.4%)	
-Insulin pump PLGS (*n*, %)	32 (18.4%)	19 (31.7%)	0 (0%)	12 (40.0%)	1 (2.6%)	

MDI, multiple dose injection; PLGS, predictive low glucose suspend system.

ns, no statistical differences.

* *p* < 0.05; ***p < 0.001.

^a^ Comparisons between AID systems.

**Table 2:** Follow-up glycemic control in all patients.

Glucometric variable	Basal	3 months	6 months	12 months	*p*
Glucose (mmol/L)	8.82 ± 1.27	8.17 ± 0.87	8.25 ± 1.02	8.27 ± 0.86	<0.001
GMI (mmol/mol)	54.3 ± 6.2	51.0 ± 3.9	51.2 ± 4.1	51.3 ± 4.0	<0.001
CV (%)	37.29 ± 6.00	32.83 ± 6.07	32.76 ± 5.33	32.91 ± 5.59	<0.001
TAR2	9.28 ± 8.43	4.86 ± 5.13	5.05 ± 4.96	5.25 ± 4.96	<0.001
TAR1	22.63 ± 8.22	17.48 ± 6.42	17.40 ± 6.45	18.06 ± 6.77	<0.001
TIR	64.22 ± 13.33	75.28 ± 9.67	75.10 ± 9.78	74.04 ± 9.59	<0.001
%TIR > 70%	28.7	71.3	70.31	60.9	0.009
TBR1	3.36 ± 2.55	2.08 ± 1.57	2.02 ± 1.72	2.18 ± 2.43	<0.001
TBR2	0.55 ± 0.98	0.36 ± 0.68	0.40 ± 0.79	0.46 ± 0.92	0.031
Objective target (%)	9.72%	53.33%	54.84%	52.07%	<0.001

Objective target: percentage of patients meeting all the international targets: TIR >70%, TAR <25%, TBR <4% and CV <36%.

Data expressed as the mean ± standard deviation (SD) for numerical variables and as a percentage for categorical variables.

98 patients (56.3%) were insulin pump users before switching and 18.4% were previous PLGS (predictive low glucose suspend) users. In terms of glycaemia control, at baseline, mean HbA1c was 55 mmol/mol, and only 42.5% of participants have levels lower than 53 mmol/mol, while after one year mean HbA1c was significantly reduced to 50 mmol/mol (*p* < 0.001), and 67.5% of participants presented levels lower than 55 mmol/mol Regarding TIR, it was improved by 10 points from the baseline (64%) to the end of the study (74%), and the proportion of patients with TIR >70% increased from 28.7% at baseline to 71.3% after three months, decreasing to 60.9% after 12 months.

Also, as observed in Table 2, glucose levels, GMI and CV were significantly improved after one year use of an AID system, however, it is important to highlight that this improvement is achieved during the first 3 months and maintained over time. Similarly, the percentage of patients achieving the consensus target (TAR <25%; TIR >70%; TBR <4%; CV <36%) remained consistent from the 3-month to the 12-month follow-up.

### Differences between systems

Among the 174 participants in the study, 4 different systems were used: 60 with MM780G, 45 with DBLG, 30 with Control-IQ and 39 with Cam-APS. At baseline, significant differences between systems were observed across several glucometric parameters (Supplementary Material Table 1), including glucose, diabetes duration and the previous treatment. Specifically, the use of previous insulin pump was significantly higher (87%) in MM780G-group and lower (59%) in CAM-APS group and DLBG system users had significantly longer duration of diabetes than the other groups. Additionally, baseline glucose control was significantly better in the MM780G group than in the other groups, as indicated by GMI, TIR, TAR and CV, though no differences were observed in TBR across the various AID systems.

Given these findings, a repeated measures ANCOVA was conducted to assess the impact of these baseline differences on glycemic outcomes. The analysis revealed that only baseline glucose levels had a significant influence on both overall glycemic control and its evolution over time. In contrast, neither diabetes duration nor previous treatment significantly affected the trajectory of glycemic improvement.

**Figure 1:**
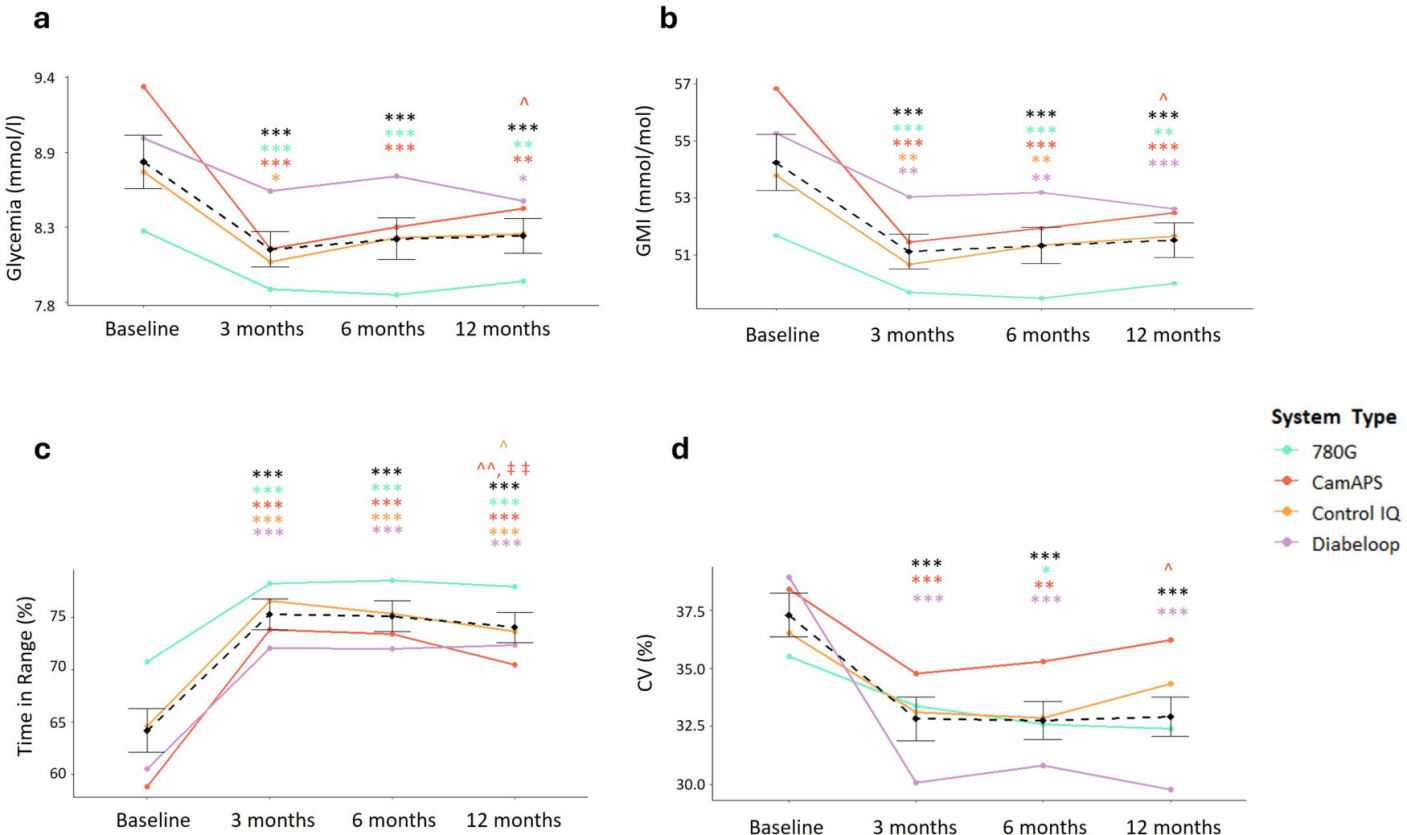
12 months effect of an AID system implementation in glucometric variables. **(a)** changes in glycaemia measured in mmol/mol. **(b)** Changes in the percentage of Glucose Management Indicator (GMI) measured in mmol/mol; **(c)** changes in the percentage of time in range (TIR); **(d)** changes in the percentage of the coefficient of variation (CV). Black lines represent the average of each value in all systems while different systems are highlighted in colors as stated in the legend. Comparisons against basal values: * *p* < 0.05; ** *p* < 0.01; *** *p* < 0.001. Comparisons against 3 months: ^ *p* < 0.05, ^^*p* < 0.01. Comparisons against 6 months: ‡ *p* < 0.01.

Regarding glycemic changes (Figure 1a and Supplementary Material Table 2**)**, a significant reduction after 12 months of AID use was observed in MM780G, CamAPS and DBLP users. In MM780G and CamAPS users the change was achieved already after 3 months and maintainded until 12 months, while in DBLP users it was only achieved after a year. By contrast, Control IQ users presented a significant reduction after 3 months, but this reduction was not maintained during further follow-up. GMI and TIR percentages were significantly reduced in all AID systems after 3 months and this reduction maintained over the 12 months of the study (Figures 1b,c and Supplementary Material Table 2). More differences were observed in the CV, where only the DBLP system users could achieve a significant reduction after 12 months (Figure 1c and Supplementary Material Table 2).

**Figure 2:**
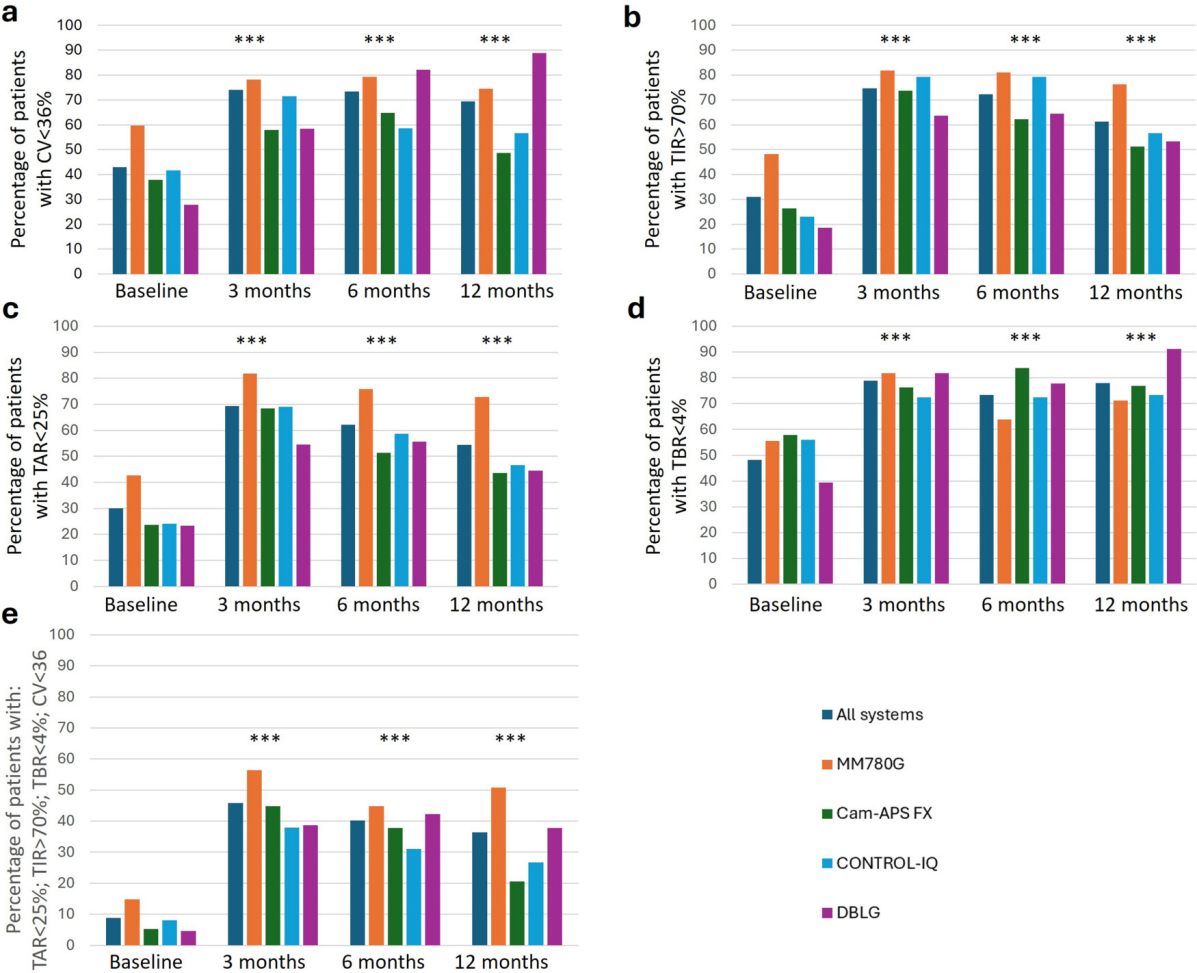
Comparative of glycemic control between different systems basal and follow up. **(a)** Percentage of patients with a coefficient of variation less than 36%; **(b)** Percentage of patients with a TIR higher than 70%: **(c)** percentage of patients with a TAR lower than 25%; **(d)** percentage of patients with a TBR lower than 4%; **(e)** percentage of patients with an optimal control according to the International consensus: TBR (time with glucose values <3.9 mmol/L) <4%; TIR >70% and TAR (time with glucose values >10 mmol/L) <25% and CV <36%. Comparisons against basal values in all groups of systems (dark blue line): * *p* < 0.05; ** *p* < 0.01; *** *p* < 0.001.

As shown in (Figure 2 and Supplementary Material Table 3), after 12 months of AID implantation, only the DBLG system achieved significant improvement in all consensus targets (TBR <4%, TIR >70%, TAR <25%, and CV <36%) individually and overall. However, initially, less than 10% of participants met the overall target, except for MM780G users, 16% of whom achieved it. After three months, 50% of MM780G and Cam-APS users, as well as approximately 40% of Control-IQ and DBLG users, had achieved the goal. However, these percentages dropped slightly, though not significantly, at 12 months for all systems except MM780G, where 50.8% of participants achieved the desired goal (Figure 2e).

## Discussion

This study demonstrates the efficacy of AIDs in treating patients with T1D, with some differences observed between the four marketed algorithms. Although the efficacy of AID systems has been reported in various randomized (10, 22–26) or real-life studies (27–29), in pediatric, adolescent and adult populations, the analysis has been limited to small cohorts and individual AID systems. These studies are not only limited to an improvement in glucometric parameters, but they have also been reported to improve other factors such as disease burden, diabetes-associated anxiety, and sleep quality (30). Initially these benefits were observed in pediatric and adolescent populations and later in adults as well. For instance, Medtronic recently published data in adults and young people, demonstrating an increase in TIR of over 75% after one month of starting MM780G, which was maintained for the first year (27).

Our sample shows a similar phenomenon, with a significant increase in TIR after 12 months of the implementation of an AID system, which is achieved at 3 months, regardless of the system used, with the exception of the Cam-APS system where TIR values decrease from the first 3 months to 1 year. This confirms the usefulness of different systems in achieving better metabolic control in the short and long term. However, there is limited experience comparing various algorithms. Bassi et al, compared the results of treating an Italian cohort of 31 children and adolescents with Control IQ or MM780G. After 1 month of treatment, they observed a significant increase in TIR and showed better glycemic control with MM780G (16). The same group, including adults, compared these 2 systems for 1 year, confirming the better response in the MM780G group (17). Similarly, Schütz et al. compared the efficacy of MM780G vs. OS-AID (Open-Source Automated Insulin Delivery) systems over a period of 3–6 months and observed a higher TIR in users of OS-AID systems (18). Differences in patient inclusion or different follow-up time could explain the differences in results in these real-life studies.

Recently, Beato-Víbora et al. conducted a prospective multicenter study comparing the efficacy of the Control IQ system vs. MM780G in adolescent and adult patients with T1D. After 3 months of follow-up, they observed an increase of 14% in TIR with no differences between the 2 systems (31). These data are consistent with those of our study, which shows a 10-point increase in TIR after 12 months of AID system implementation, independently on the different system used. It is important to highlight that, from the various glucometric variables analyzed, TBR is of great importance since hypoglycemia is the most dangerous event for people with diabetes, especially when it occurs at night, being AID systems a very useful tool to prevent these episodes (32). After 12 months of use, more than 70% of people have achieved TBR targets of less than 4%, regardless of the system. In contrast, fewer people achieve CV, TIR, and TAR targets after 12 months. The enhanced TBR in comparison to the other metrics can be attributed to the fact that AID systems are equipped with an automated insulin suspension feature that responds to low glucose levels.

Additionally, we measured the glycaemic control according to the International Consensus targets (TAR <25%; TIR >70%; TBR <4%; CV <36%) and observed an increase of more than 40 percentual points after 12 months (from 9.7% to 52%).

We also observed differences between the systems. Although no significant differences were observed after 3 months, when the percentage of patients achieving the aforementioned target increased between 33 and 42 points, we observed a superiority in the MM780G group after one year of treatment, with up to 50% of patients achieving optimal glycaemic control. In contrast, although 50% of patients in the Cam-APS group achieved the target at three months, only 28.7% maintained this control at 1 year. This is consistent with the previously described comparative studies showing superiority of the MM780G systems. However, further studies are needed to confirm this evidence.

Regarding the limitations of the study, it should be noted that it was a descriptive design of our typical clinical practice. Additionally, the group of patients using MM780 system had better control prior to the start of the study. This may be attributed to the fact that 31.7% of these patients were already using a PLGS system, which could be advantageous for this group, although the statistical analysis took these baseline differences into account. This is in line with previous studies that have demonstrated an increase in TIR in patients who switch from PLGS to AID systems without an increase in the risk of hypoglycemia (33).

To obtain a comprehensive evaluation of these systems, it is important to consider other related endpoints, such as the difficulty of management or quality of life. Although positive experiences have been reported regarding the quality of life of relatives of pediatric patients (34), it is important to maintain objectivity and avoid making subjective evaluations. In this respect, Navas et al. conducted a retrospective study comparing the efficacy of MM780G, DLBG1 and Control IQ in 75 users over a period of 6 months. They observed an improvement in the patients' quality of life but did not find a correlation with improved glycemic control (35). This suggests that other factors related to the use of these systems should be considered when recommending a particular system.

To make a proper comparison, further studies are required with a larger sample size and longer follow-up to confirm the sustained effect on glycemic control, quality of life, and the development of complications over time. On the other hand, randomized trials are needed to compare the differences between the different systems to determine which system is best for each patient.

The difference in the number of patients in each group is probably due to the lack of availability of these systems (where mainly Medtronic and Tandem systems could be used) or the delay in the hospital care in the first year post-pandemic. Nevertheless, this sample provides valuable insights that reflect routine clinical practice. In addition, we have a cohort of patients followed for 1 year with a low dropout rate.

In conclusion, AID systems have proven to be an effective tool in the management of patients with T1D. Today, we have different algorithms that have been shown to be comparable in their efficacy. This opens the possibility of personalizing the treatment of patients with T1D. However, further studies are needed to determine which patient profile is best suited to be improved by each of these systems.

## Data Availability

The original contributions presented in the study are included in the article/Supplementary Material, further inquiries can be directed to the corresponding author.
