# Supplementary material for: One-year evaluation of automated insulin delivery systems in adults with type 1 diabetes
Source: Front Digit Health. 2025 Jul 1;7:1596188. doi: 10.3389/fdgth.2025.1596188 (PMC12259683; doi:10.3389/fdgth.2025.1596188)
Supplement: Supplementary file 1 [file Datasheet1.pdf]

Supplementary Table 1 Basal and follow-up glucometric differences between systems

BASAL	MM780G	Cam-APS FX	CONTROL-IQ	DBLG	p
**Glucose (mmol/L)**	8.31 ± 0.73	9.38 ± 1.59	8.75 ± 1.28	9.00 ± 1.26	<0.001
**HbA1c (mmol/mol)**	53.6 ± 7.5	59.2 ± 10.4	53.7 ± 9.2	56.5 ± 9.2	0.053
**GMI (mmol/mol)**	51.6 ± 3.4	53.6 ± 6.1	56.8 ± 7.5	55.2 ± 6.6	<0.001
**CV (%)**	34.49 ± 5.22	38.41 ± 5.91	36.53 ± 5.30	38.92 ± 6.79	0.022
**TAR2**	5.46 ± 4.13	13.34 ± 11.24	8.84 ± 7.45	10.72 ± 8.30	<0.001
**TAR1**	20.18 ± 7.00	24.24 ± 8.52	23.20 ± 9.49	23.93 ± 8.17	0.058
**TIR**	70.74 ± 8.59	58.84 ± 15.53	64.58 ± 13.71	60.56 ± 12.82	<0.001
**TBR1**	3.13 ± 2.27	3.16 ± 2.52	2.92 ± 2.53	4.09 ± 2.83	0.172
**TBR2**	0.5 ± 0.86	0.43 ± 1.00	0.48 ± 0.87	0.74 ± 1.14	0.469
**3 months**					
**Glucose (mmol/L)**	7.87 ± 0.70	8.17 ± 0.91	8.08 ± 0.89	8.60 ± 0.85	<0.001
**HbA1c (mmol/mol)**	50.9 ± 6.7	47.9 ± 6.6	48.1 ± 4.4	46.9 ± 5.8	0.323
**GMI (mmol/mol)**	49.7 ± 3.3	51.4 ± 3.7	50.6 ± 4.4	53.0 ± 3.8	<0.001
**CV (%)**	33.39 ± 7.67	34.77 ± 4.79	33.11 ± 5.60	30.07 ± 3.86	0.004
**TAR2**	3.26 ± 3.80	5.61 ± 5.03	4.38 ± 4.75	6.52 ± 6.28	0.01
**TAR1**	15.8 ± 6.81	18.24 ± 5.95	16.52 ± 6.41	19.55 ± 5.80	0.022
**TIR**	78.18 ± 8.50	73.82 ± 10.12	76.55 ± 9.98	72.07 ± 9.50	0.01
**TBR1**	2.42 ± 1.92	2.24 ± 1.20	2.10 ± 1.68	1.50 ± 1.13	0.029
**TBR2**	0.35 ± 0.80	0.18 ± 0.39	0.55 ± 0.78	0.41 ± 0.61	0.16
**6-months**					
**Glucose (mmol/L)**	7.82 ± 0.78	8.33 ± 0.80	8.26 ± 0.94	8.71 ± 1.29	<0.001
**HbA1c (mmol/mol)**	50.4 ± 6.3	47.5 ± 5.3	47.9 ± 5.1	47.9 ± 6.0	0.452
**GMI (mmol/mol)**	49.4 ± 3.7	51.8 ± 3.8	51.3 ± 4.4	53.2 ± 4.1	<0.001
**CV (%)**	32.60 ± 4.65	35.28 ± 5.02	32.28 ± 5.02	32.85 ± 6.48	0.002
**TAR2**	3.31 ± 3.75	6.19 ± 5.10	5.31 ± 4.54	6.18 ± 5.92	0.008
**TAR1**	15.24 ± 6.41	18.16 ± 5.87	17.00 ± 6.95	19.82 ± 5.82	0.003
**TIR**	78.50 ± 8.34	73.41 ± 9.83	75.31 ± 10.6	71.98 ± 9.83	0.005
**TBR1**	2.52 ± 2.11	1.84 ± 1.01	2.03 ± 1.66	1.51 ± 1.52	0.025
**TBR2**	0.43 ± 0.88	0.41 ± 0.93	0.28 ± 0.53	0.44 ± 0.69	0.816
**1-year**					
**Glucose (mmol/L)**	7.94 ± 0.76	8.47 ± 0.86	8.28 ± 1.01	8.53 ± 0.75	0.001
**HbA1c (mmol/mol)**	51.3 ± 5.3	47.9 ± 7.2	48.4 ± 7.0	49.1 ± 8.1	0.303
**GMI (mmol/mol)**	49.9 ± 3.5	52.3 ± 4.1	51.6 ± 4.8	52.4 ± 3.5	0.003
**CV (%)**	32.40 ± 5.68	36.22 ± 4.07	34.32 ± 6.68	29.78 ± 3.80	<0.001
**TAR2**	4.03 ± 4.92	6.69 ± 5.65	5.90 ± 5.55	5.15 ± 3.48	0.059
**TAR1**	15.51 ± 6.15	19.28 ± 6.86	17.60 ± 6.73	20.64 ± 6.45	<0.001
**TIR**	77.90 ± 8.87	70.44 ± 9.94	73.63 ± 10.12	72.36 ± 8.26	<0.001
**TBR1**	2.22 ± 1.90	3.08 ± 3.94	2.13 ± 1.83	1.38 ± 1.10	0.015
**TBR2**	0.29 ± 0.65	0.51 ± 1.25	0.73 ± 1.23	0.47 ± 0.59	0.188

Supplementary Table 2. Glucometric statistical differences between systems (p-value)

	Baseline vs 3 Months	Baseline vs 6 Months	Baseline vs 12 Months	3 months vs 6 months	3 months vs 12 months	6 months vs 12 months
**MG789**						
**Glucose**	<0.001	<0.001	0.001	1.000	1.000	1.000
**GMI**	<0.001	<0.001	0.003	1.000	1.000	1.000
**CV**	0.920	0.037	0.110	1.000	1.000	1.000
**TIR**	<0.001	<0.001	<0.001	1.00	1.00	1.000
**TAR2**	0.002	0.002	0.089	0.730	0.170	0.170
**TAR1**	<0.001	<0.001	<0.001	1.000	1.000	1.000
**TBR1**	1.000	1.000	0.900	1.000	1.000	1.000
**TBR2**	0.797	1.000	0.374	0.797	1.00	0.797
**Cam-APS**						
**Glucose**	<0.001	<0.001	0.004	0.107	0.011	0.079
**GMI**	<0.001	<0.001	<0.001	0.161	0.041	0.161
**CV**	<0.001	0.009	0.102	0.189	0.010	0.159
**TIR**	<0.001	<0.001	<0.001	0.382	0.007	0.009
**TAR2**	<0.001	<0.001	<0.001	0.167	0.020	0.167
**TAR1**	<0.001	<0.001	0.062	0.896	0.759	0.759
**TBR1**	0.069	0.012	0.837	0.174	0.402	0.290
**TBR2**	0.423	1.000	1.000	0.493	0.633	1.000
**Control IQ**						
**Glucose**	0.017	0.059	0.059	0.917	0.191	0.917
**GMI**	0.007	0.001	0.063	1.000	1.000	1.000
**CV**	0.053	0.070	0.744	0.867	0.053	0.053
**TIR**	<0.001	<0.001	<0.001	0.463	0.031	0.463
**TAR2**	0.025	0.088	0.124	0.469	0.189	0.533
**TAR1**	0.006	0.136	0.004	0.485	0.875	0.875
**TBR1**	0.377	0.672	0.840	0.672	0.377	0.840
**TBR2**	1.000	1.000	1.000	1.000	1.000	1.000
**Diabeloop**						
**Glucose**	0.213	0.500	0.015	0.885	0.885	0.885
**GMI**	0.001	0.001	<0.001	0.898	0.565	0.565
**CV**	<0.001	<0.001	<0.001	0.202	0.833	0.126
**TIR**	<0.001	<0.001	<0.001	1.000	1.000	1.000
**TAR2**	<0.001	<0.001	<0.001	0.887	0.463	0.463
**TAR1**	0.056	0.024	0.025	1.000	1.000	1.000
**TBR1**	<0.001	<0.001	<0.001	0.241	0.849	0.234
**TBR2**	0.245	0.681	9,607	0.594	0.681	0.681

p-value adjusted by previous use of insulin pump, diabetes duration and basal glucose

Supplementary Table 3. Cochrane Q test P-values from Figure 2

	Baseline vs 3 Months	Baseline vs 6 Months	Baseline vs 12 Months	3 months vs 6 months	3 months vs 12 months	6 months vs 12 months
**All systems**						
**CV**	<0.001	<0.001	<0.001	0.728	0.224	0.501
**TIR**	<0.001	<0.001	<0.001	1.000	0.249	0.098
**TBR**	<0.001	<0.001	<0.001	0.096	0.585	0.414
**TAR**	<0.001	<0.001	<0.001	0.185	0.005	0.088
**Objetive**	<0.001	<0.001	<0.001	0.560	0.413	0.560
**MG789**						
**CV**	0.172	0.172	0.237	1.000	1.000	1.000
**TIR**	<0.001	<0.001	<0.001	1.000	0.399	0.400
**TBR**	0.014	0.508	0.178	0.035	0.178	0.508
**TAR**	<0.001	<0.001	<0.001	0.727	0.400	0.609
**Objetive**	<0.001	0.005	<0.001	0.845	0.845	0.516
**Cam-APS**						
**CV**	ns	ns	ns	ns	ns	ns
**TIR**	0.045	<0.001	0.039	0.832	0.832	0.328
**TBR**	0.147	0.044	0.237	0.750	0.754	0.328
**TAR**	<0.001	<0.001	<0.001	0.059	0.026	0.549
**Objetive**	0.004	0.004	0.078	0.688	0.086	0.216
**Control IQ**						
**CV**	0.234	0.434	0.544	0.434	0.375	1.000
**TIR**	0.015	<0.001	0.003	0.659	1.000	0.563
**TBR**	ns	ns	ns	ns	ns	ns
**TAR**	0.023	0.023	0.125	1.000	0.544	0.544
**Objetive**	0.021	0.023	0.031	0.544	0.544	1.000
**Diabeloop**						
**CV**	<0.001	<0.001	<0.001	1.000	1.000	1.000
**TIR**	<0.001	<0.001	<0.001	0.545	0.545	1.000
**TBR**	<0.001	<0.001	<0.001	0.688	0.150	0.047
**TAR**	0.004	0.004	0.004	1.00	0.905	0.823
**Objetive**	<0.001	<0.001	<0.001	0.949	1.000	0.949

Objetive target: Percentage of patinets meeting all the international targets: TIR>70%, TAR<25%, TBR<4% and CV<36%
